# Evaluation of the relationship between left atrial stiffness, left ventricular stiffness, and left atrioventricular coupling index in type 2 diabetes patients: a speckle tracking echocardiography study

**DOI:** 10.3389/fcvm.2024.1372181

**Published:** 2024-04-25

**Authors:** Hai Nguyen Ngoc Dang, Thang Viet Luong, Binh Anh Ho

**Affiliations:** ^1^ The Faculty of Medicine, Duy Tan University, Da Nang, Vietnam; ^2^ Department of Internal Medicine, Hue University of Medicine and Pharmacy, Hue, Vietnam; ^3^ Cardiovascular Center, Hue Central Hospital, Hue, Vietnam

**Keywords:** speckle tracking echocardiography, left atrial stiffness, left ventricular stiffness, left atrioventricular coupling index, diabetes mellitus

## Abstract

**Background:**

Cardiovascular complications are a leading cause of mortality and disability in individuals with diabetes mellitus (DM). Moreover, DM can directly impact the structure and function of cardiac muscle. We conducted a study to evaluate cardiac stiffness in DM patients in both the left atrium (LA) and left ventricle (LV), as well as to assess the impact of DM on the synchronization of the LA and LV, particularly within the Vietnamese population, utilizing speckle tracking echocardiography (STE).

**Methods:**

We studied 111 research subjects divided into two groups comprising 52 patients with DM and 59 healthy individuals. All the subjects provided relevant clinical information, and echocardiography was performed to assess the indices of LA stiffness, LV stiffness, and left atrioventricular coupling index (LACI).

**Results:**

Our study indicated that DM patients exhibited greater LA and LV stiffness than control patients. The LACI (%) in the DM group was also greater than that in the control group (17.12% ± 6.72% vs. 12.28% ± 3.96%, respectively; *p* < 0.001). The LACI was positively correlated with the LA and LV stiffness indices. Decreased levels of LV GLS, adjusted for age, sex, blood pressure, and BMI, have emerged as identified risk factors for DM.

**Conclusions:**

LA stiffness, LV stiffness, and the LACI are greater in DM patients than in normal individuals.

## 1 Introduction

Diabetes mellitus (DM), particularly type 2 DM, is a prevalent medical condition. As of 2021, approximately 1 in 10 adults worldwide have DM. The global prevalence of DM is on the rise, with an estimated 783.2 million individuals (12.2%) projected to have DM, especially in developing countries such as Vietnam, where the trend is increasing (1).

Cardiovascular complications are a leading cause of mortality and disability in individuals with DM (2). DM increases the risk of cardiovascular diseases such as hypertension, coronary artery disease, and heart failure. Moreover, DM can directly impact the structure and function of cardiac muscle (3, 4). Despite recent advancements, effective therapeutic approaches for DM-related cardiac dysfunction remain limited. Cardiac dysfunction often remains clinically silent in DM patients and is frequently undetected until the later stages of the disease. Even in patients without symptoms, normal blood pressure, and well-controlled DM (5), the silent increase in blood glucose impedes optimal cellular glucose utilization, leading to cardiac fibrosis. Cardiac fibrosis, characterized by increased extracellular matrix proteins, collagen deposition, disruption of cardiac cell order, and restructuring of the cardiac architecture, is a hallmark of DM-related cardiovascular changes (2). Changes in cardiac structure due to DM-related fibrosis are often associated with alterations in myocardial stiffness (6).

Cardiovascular complications in DM patients are typically investigated only after the manifestation of evident heart failure symptoms. With advanced imaging techniques and increasing awareness that heart failure is becoming more prevalent in DM patients, early detection of cardiac fibrotic changes is possible. Cardiac magnetic resonance imaging is widely considered the gold standard for evaluating cardiac function. However, cardiac magnetic resonance imaging is not always readily available, especially in remote or economically disadvantaged areas (7, 8). Similarly, myocardial perfusion imaging in laboratory animals provides high-resolution imaging but is impractical for continuous monitoring in a clinical setting. Recent advancements in echocardiographic imaging techniques, particularly speckle tracking echocardiography (STE), have allowed continuous, detailed, noninvasive, and relatively high-resolution assessments of cardiac function in both clinical and experimental settings, overcoming limitations in time and accessibility (5). Among these newer ultrasound techniques, STE has emerged as a noninvasive biomarker for the early detection of morphological and functional changes in the heart (9).

The stiffness of the left atrium (LA) and left ventricle (LV) has been demonstrated to be a crucial index for assessing and predicting various conditions, such as hypertension, atrial fibrillation, coronary artery disease, and heart failure (10–14). Additionally, the left atrioventricular coupling index (LACI) is considered one of the indices that can predict the likelihood of atrial fibrillation, heart failure, and other cardiovascular events early in life (15–17). Recognizing the significant role of LA stiffness, LV stiffness, and LACI, we hypothesize that DM may influence the stiffness of the LV and LA and the synchronization of the LA and LV in diabetic patients. Therefore, we conducted a study to assess cardiac stiffness in DM patients in both the LV and LA, as well as the impact of DM on the synchronization of the LA and LV, particularly in the Vietnamese population, where there is an increasing trend in DM incidence (1), using STE.

## 2 Materials and methods

### 2.1 Study population

A cross-sectional study was conducted at the University Medical Hospital in Hue from February 2022 to October 2023. The study was approved by the Institutional Review Board of Hue University of Medicine and Pharmacy (No: H2022/312). The study participants were adults aged 18 years and older who provided informed consent before enrollment.

We divided the study population into two groups: the type 2 DM group and the healthy control group. The type 2 DM group comprised 52 consenting adults who met the selection criteria and did not meet the exclusion criteria. These patients were diagnosed with diabetes following the guidelines of the American Diabetes Association and the European Association for the Study of Diabetes (18). The exclusion criteria included hypertension, severe valvular heart disease, congenital heart disease, hypertrophic cardiomyopathy, dilated cardiomyopathy, ischaemic heart disease, reduced ejection fraction heart failure, atrial fibrillation, pacemaker implantation, other severe cardiac arrhythmias, severe comorbidities affecting life expectancy, insufficient echocardiographic data, and poor-quality echocardiographic images for analysis. The control group consisted of 59 healthy adults who underwent health screening, had no history of cardiovascular disease or diabetes, and were age-matched to the type 2 DM group.

### 2.2 Clinical data collection

The clinical data collected included personal and family medical histories and clinical variables obtained through direct interviews and medical records. Patient measurements included height (measured to the nearest 0.1 centimeters), weight (measured to the nearest 0.1 kilograms), and waist circumference (WC) (measured to 0.1 centimeters). Body mass index (BMI) was calculated as weight in kilograms divided by the square of height in meters (19). Body surface area (BSA) was calculated using the formula BSA = (1/6)(Weight*Height)^0.5^ (20). Blood pressure was determined by the mean of three appropriately sized automated cuff readings taken one minute apart after five minutes of rest. Laboratory data, including glycated haemoglobin (HbA1c) levels, were collected. HbA1c levels were measured using a Roche analyser.

### 2.3 Transthoracic echocardiogram

We utilized a specialized Affiniti 70 echocardiography machine from Philips Healthcare at the University Medical Hospital in Hue. During the ultrasound procedure, the machine simultaneously recorded the electrocardiogram alongside the echocardiographic images. All echocardiograms included in the final data analysis were performed on patients in normal sinus rhythm. The echocardiographic procedure adhered to the guidelines outlined by the American Society of Echocardiography for performing a comprehensive transthoracic echocardiographic examination in adults (21). These measurements are performed by an echocardiography expert, who is independent of the research team and is unaware of other patient information during processing.

### 2.4 Speckle tracking echocardiography

During the ultrasound examination, patients were routinely imaged in the apical four-chamber, two-chamber, and three-chamber views. Each image was acquired over two consecutive cardiac cycles and subsequently stored on a USB drive. The speckle tracking analysis of myocardial strain was performed using offline QLAB software version 15.

To assess LV strain, a cardiac cycle was defined from the *R* wave to the *R* wave. The selection of end-diastolic and end-systolic frames was based on the closure of the mitral and aortic valves, respectively. At the mitral annulus, two reference points were positioned, and one was placed at the apex. The process involved automated border tracing, with potential manual adjustments if deemed necessary. LV global longitudinal strain (GLS) was then calculated using three apical chamber views. Individuals with compromised image quality, insufficient border tracking, shortened views, or missing images were excluded from the analysis.

LA strain was assessed in both the two-chamber and four-chamber views using reference points beginning at the *P* wave and *R* wave in the cardiac cycle. The average strain values were calculated from two references. LA strain measurements were obtained during the reservoir, conduit, and contractile phases of LA function, denoted as the LA strain reservoir function (LASr), LA strain conduit function (LAScd), and LA strain contractile function (LASct), respectively.

**Figure 1:**
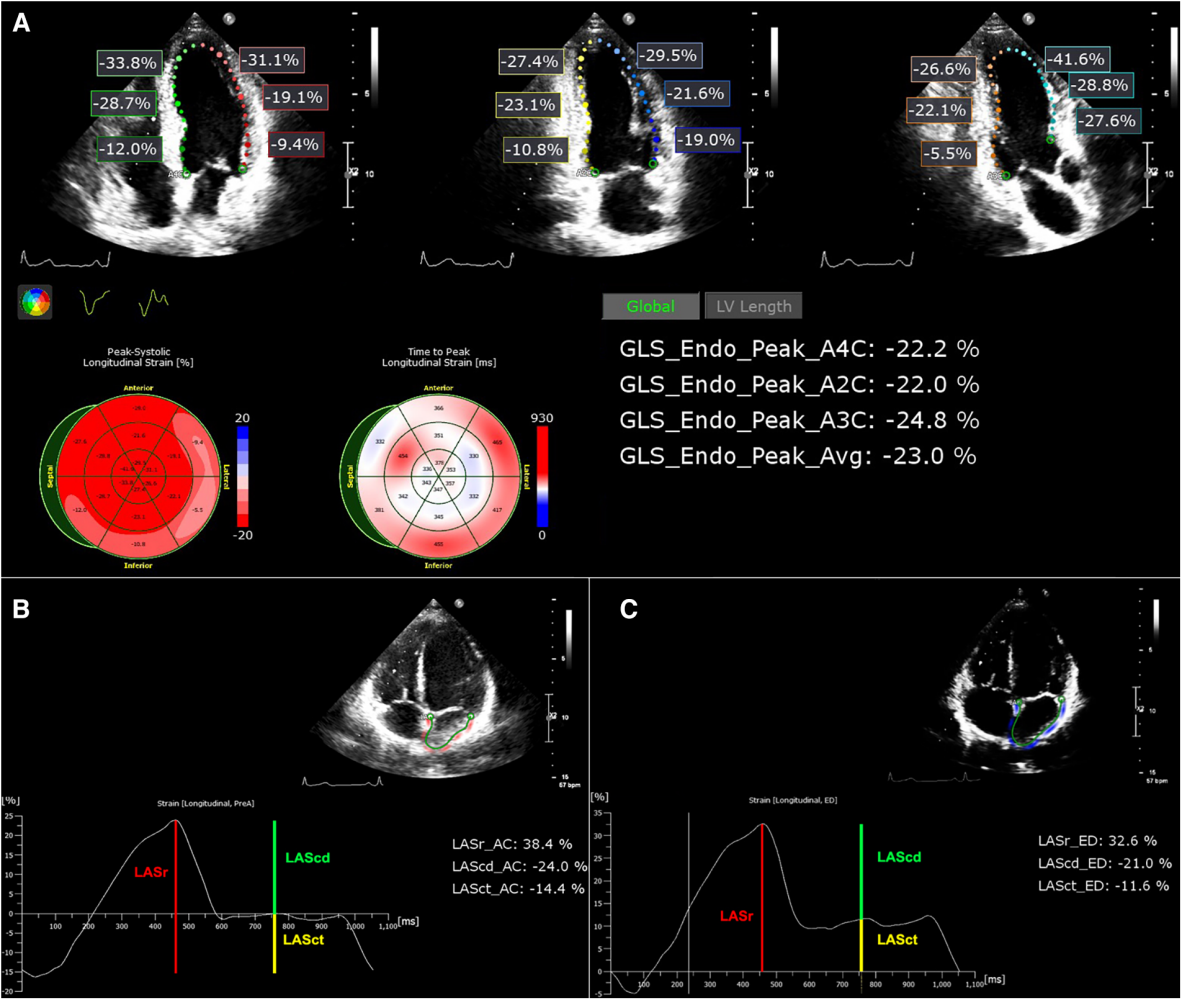
(**A**) Images of LV strain analysis. The results are displayed with the LV GLS for the apical 4-chamber, 2-chamber, and 3-chamber chambers and their respective averages. (**B,C**) LA strain images with reference points beginning at the *P* wave and *R* wave, respectively.

The results of the LV and LA strains are conventionally represented as negative values. However, for convenience in analysis and display, we utilized the absolute values of these results. These parameters were measured by an independent echocardiography specialist who was not affiliated with the research team and remained uninformed about other patient details throughout the procedure. The detailed methodology is illustrated in Figure 1.

### 2.5 Left atrial stiffness

The initial report introducing the concept and validating the noninvasive estimation of LA stiffness was by Kurt et al. (22). The LA stiffness is calculated as the ratio of E/e′ to LASr. This formula has been widely employed in numerous studies to assess LA stiffness (23, 24). The LA filling index is determined by the ratio of the mitral early-diastolic inflow peak velocity (E) to the LASr (E/LASr ratio) (25, 26). Additionally, recent studies have recognized the role of the LAVI/LASr ratio as equivalent to the aforementioned measures (27).

### 2.6 Left ventricular stiffness

LV stiffness is assessed using several parameters, including diastolic wall strain (DWS), which is calculated as (LV posterior wall thickness at systole—LV posterior wall thickness at diastole)/LV posterior wall thickness at systole (28–30). Additionally, LV stiffness can be evaluated through the ratios of E/e′ to LV end-diastolic volume (LVEDV) and E/LASr to LVEDV (31).

### 2.7 Left atrioventricular coupling index

**Figure 2:**
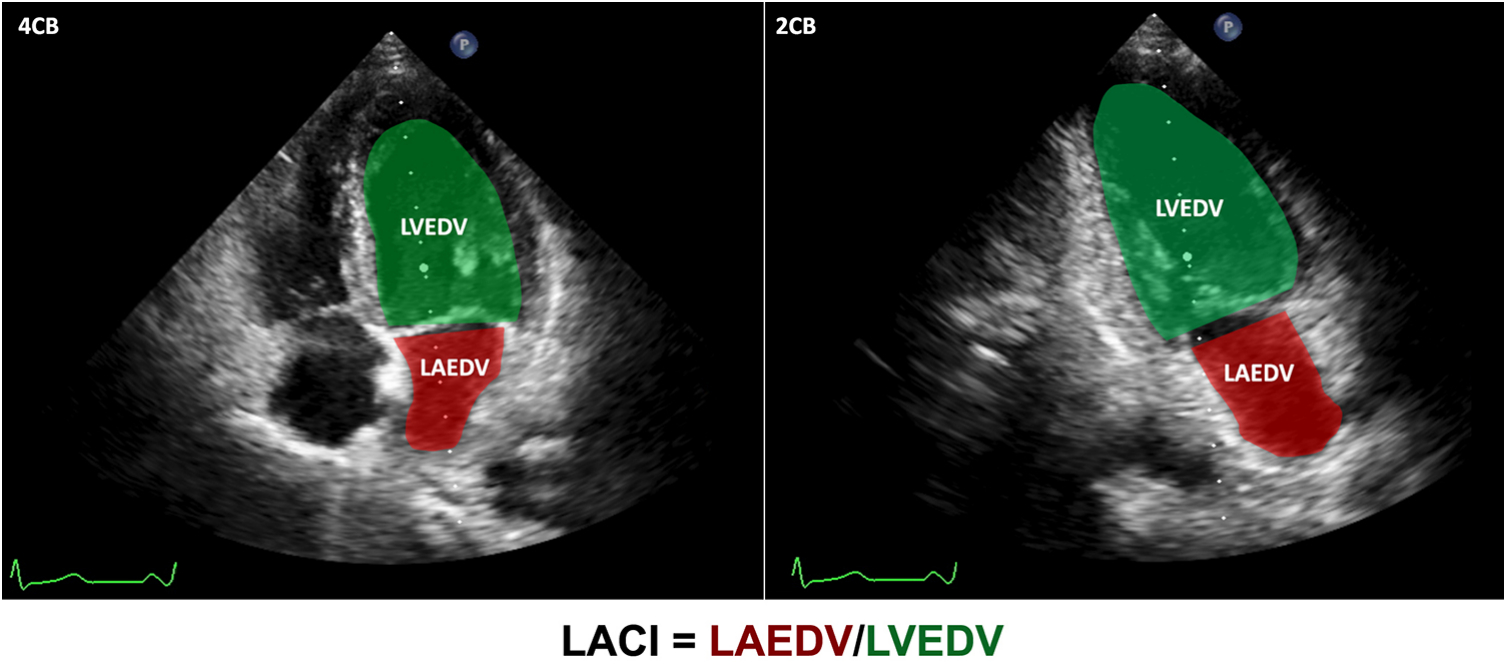
LACI calculation. LVEDV in green and LAEDV in red were measured in both the apical 4-chamber and 2-chamber views during the end-diastolic phase defined by the *R* wave on the ECG, respectively. Subsequently, the ultrasound machine automatically computed the LVEDV and LAEDV using Simpson's method. The LACI results were calculated using the formula depicted in the figure.

The LACI is defined as the ratio between the LA end-diastolic volume (LAEDV) and the LV end-diastolic volume (LVEDV). LA and LV volumes are measured at the same end-diastolic phase defined by the *R* wave on the ECG. LACI values are expressed as a percentage, where a higher LACI indicates a greater disproportion between LA and LV volumes at the end-diastole, reflecting a more significant impairment of LACI (17). Figure 2 provides an example of how this index is determined. The parameters LVEDV and LAEDV are also measured during the echocardiography process and are measured by an independent echocardiography specialist who is not affiliated with the research team. This specialist remains unaware of any other patient information during the procedure.

### 2.8 Statistical analysis

The normality of the data was assessed with the Kolmogorov‒Smirnov test. Normally distributed continuous variables are presented as the means ± standard deviations, while nonnormally distributed variables are expressed as medians with interquartile ranges (IQRs) = 25th–75th percentiles. Baseline characteristics were compared between individuals with type 2 DM and those without type 2 DM. Independent *t* tests were used for the analysis of continuous variables, and the results are reported as the means ± SDs. The Mann‒Whitney *U* test was applied for nonnormally distributed variables. Categorical variables are displayed as absolute numbers (percentages) and were compared using the chi-square test.

For the evaluation of LA stiffness, LV stiffness, and LACI between the type 2 DM and non-type 2 DM groups, the independent *t* test and Mann‒Whitney *U* test were used for normally and nonnormally distributed continuous variables, respectively. Pearson's correlation coefficient (r) along with its corresponding *p* value was used for normally distributed variables to elucidate the correlation between continuous variables, while Spearman's correlation coefficient was applied for nonnormally distributed variables. Univariate logistic regression was used to calculate odds ratios (ORs) to predict DM. In multiple logistic regression analysis, all variables with *p* ≤ 0.05 were included to explore the relevance of DM. All probability values were two-sided, and *p* values less than 0.05 were considered to indicate statistical significance. SPSS Statistics version 26 (IBM, Armonk, NY, USA) was used for all calculations.

## 3 Results

### 3.1 Clinical characteristics of the study groups

In our study, when comparing the DM group and the non-DM group, there were no significant differences in age, sex, BMI, BSA, WC, diastolic blood pressure, or alcohol consumption (*p* > 0.05). Moreover, the DM group had a duration of diabetes of 6.3 ± 5.4 years, and the average HbA1c concentration was 11.18% ± 2.97%. The detailed parameters are presented in the baseline demographic and clinical features section of Table 1.

**Table 1:** Characteristics of DM patients vs. control group.

Characteristics	DM (*N* = 52)	Control (*N* = 59)	*p*-value
Baseline demographic and clinical features			
Age (years)	59.62 ± 8.19	59.39 ± 5.69	0.868
Female (%)	34 (65.4)	34 (57.6)	0.440
BMI (kg/m^2^)	22.34 ± 2.92	22.05 ± 2.04	0.542
BSA (m^2^)	1.55 ± 0.15	1.54 ± 0.12	0.673
WC (cm)	75.58 ± 9.55	75.95 ± 4.54	0.798
SBP (mmHg)	123.77 ± 9.22	120.59 ± 7.37	0.046
DBP (mmHg)	72.54 ± 8.31	74.92 ± 7.34	0.112
Smoking (%)	13 (25.0)	4 (6.8)	0.009
Alcohol consumption (%)	10 (19.2)	14 (23.7)	0.647
Duration of diabetes (years)	6.3 ± 5.4		
HbA1C (%)	11.18 ± 2.97		
LA parameters on echocardiography			
LAd (cm)	3.13 ± 0.42	3.05 ± 0.49	0.330
LAVI (ml/m^2^)	18.53 ± 6.81	18.89 ± 3.85	0.736
LASr (%)	31.52 ± 4.62	38.81 ± 5.38	<0.001
LASct (%)	16.81 ± 4.91	19.09 ± 5.39	0.021
LAScd (%)	14.62 ± 6.30	19.72 ± 5.84	<0.001
LV parameters on echocardiography			
LVMI (g/m^2^)	106.76 ± 28.66	94.12 ± 22.75	0.011
LVEF (%)	56.31 ± 3.56	56.12 ± 3.09	0.771
LV GLS (%)	18.22 ± 2.10	21.24 ± 1.74	<0.001
Doppler parameters on echocardiography			
E velocity (cm/s)	71.03 ± 20.45	66.7 ± 14.32	0.205
A velocity (cm/s)	93.27 ± 21.67	81.18 ± 15.87	0.001
e′ septal velocity (cm/s)	6.89 ± 2.71	7.49 ± 2.03	0.186
e′ lateral velocity (cm/s)	8.56 ± 3.10	9.60 ± 2.38	0.049
E/A ratio	0.79 ± 0.28	0.84 ± 0.20	0.291
E/e′ ratio	9.95 ± 2.94	8.33 ± 2.23	0.002
TRVmax (cm/s)	156.61 ± 59.75	129.33 ± 44.17	0.008

Values are presented as mean ± standard deviation or number (%). DM, diabetes mellitus; BSA, body surface area; BMI, body mass index; WC, waist circumference; SBP, systolic blood pressure; DBP, diastolic blood pressure; HbA1c, glycated hemoglobin; LVMI, left ventricular mass index; LVEF, left ventricular ejection fraction; LV GLS, left ventricular global longitudinal strain; LAd, left atrial diameter; LAVI, left atrial volume index; E, E-wave velocity; A, A-wave velocity; e′, e′-wave velocity; TRVmax, tricuspid regurgitation velocity max; LASr, left atrial reservoir function; LAScd, left atrial conduit function; LASct, left atrial contractile function.

In the evaluation of LA morphology and functional parameters via echocardiography, we observed that the LASr, LASct, and LAScd indices were significantly lower in the DM group than in the control group. Specifically, the LASr, LASct, and LAScd indices between the DM and control groups were 31.52% ± 4.62% and 38.81% ± 5.38%, 16.81% ± 4.91% and 19.09% ± 5.39%, 14.62% ± 6.3% and 19.72% ± 5.84%, respectively. Details are shown in the LA parameters in the echocardiography section of Table 1.

The LV GLS of the DM group was lower than that of the control group. Conversely, the LVMI in the DM group was greater. This difference was statistically significant. The detailed LV parameters obtained via echocardiography are shown in the LV parameters in the echocardiography section of Table 1.

Doppler echocardiography revealed that the E/e′ ratio in the DM group was significantly greater than that in the control group (*p* = 0.002). Additionally, other Doppler indices are presented in the Doppler parameters in the echocardiography section of Table 1.

### 3.2 Comparison of LA stiffness, LV stiffness, and LACI between DM and control groups

**Figure 3:**
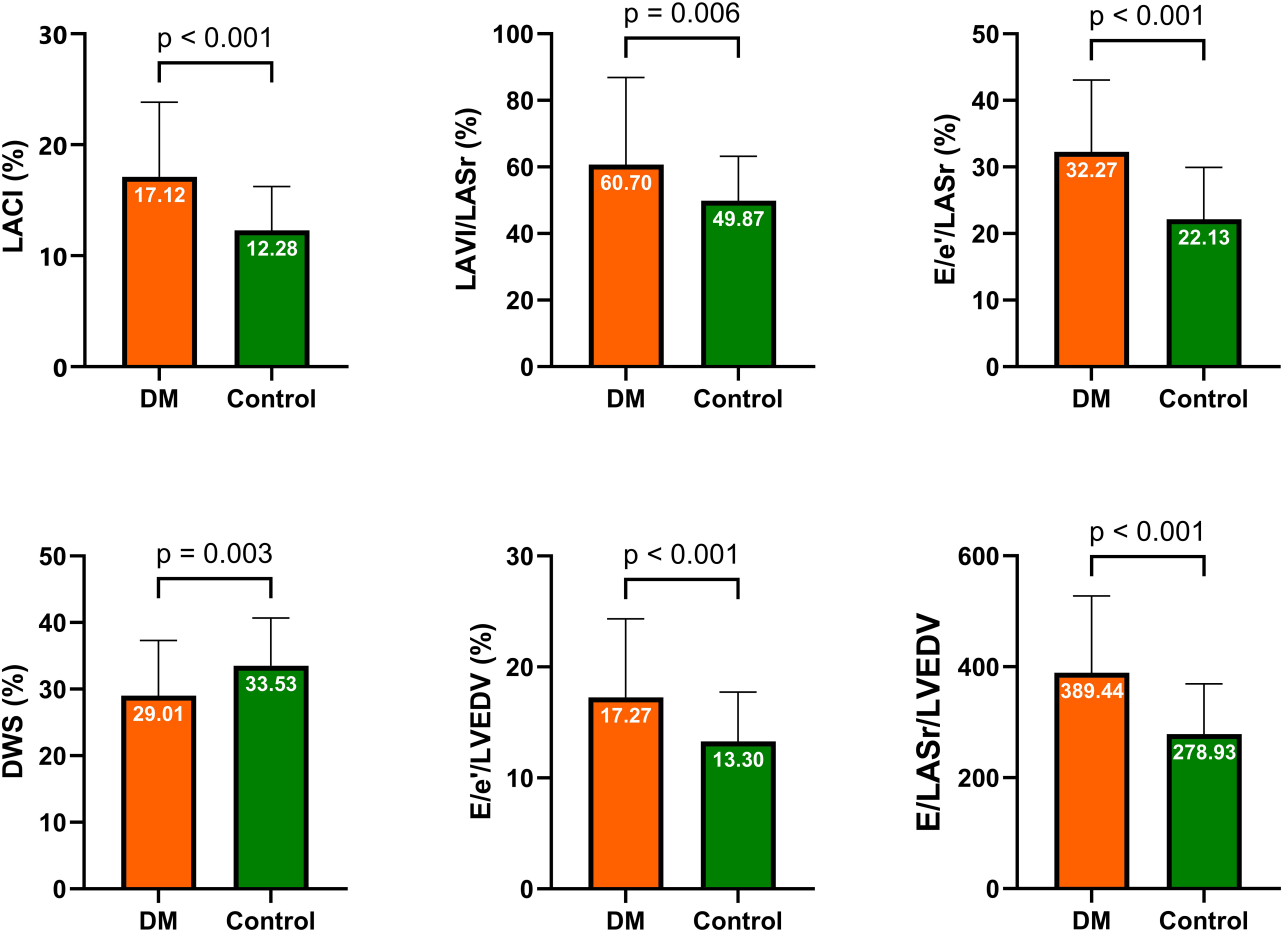
Comparison of LA stiffness indicators, LV stiffness indicators, and LACI between the DM and control groups. DM, diabetes mellitus; LA, left atrium; LV, left ventricular; LACI, left atrioventricular coupling index; DWS, diastolic wall strain.

Our study revealed greater E/e′/LASr and E/LASr ratios in the DM group than in the control group (32.27% ± 10.79% vs. 22.13% ± 7.81% and 228.23 ± 68.74% vs. 176.40% ± 52.15%, respectively), both with *p* < 0.001. Similarly, the E/LASr and LVEDV ratios, as well as the E/e′ and LVEDV ratios, were greater in the DM group than in the control group. Additionally, the LACI (%) in the DM group was also greater than that in the control group (17.12% ± 6.72% vs. 12.28% ± 3.96%, respectively; *p* < 0.001). The details are presented in Table 2 and illustrated in Figure 3.

**Table 2:** Comparison of LA stiffness indicators, LV stiffness indicators, and LACI between DM and control groups.

Characteristics	DM (*N* = 52)	Control (*N* = 59)	*p*-value
LA stiffness parameters			
E/e′/LASr ratio (%)	32.27 ± 10.79	22.13 ± 7.81	<0.001
E/LASr ratio (%)	228.23 ± 68.74	176.40 ± 52.15	<0.001
LAVI/LASr ratio (%)	60.70 ± 26.21	49.87 ± 13.35	0.006
LV stiffness parameters			
DWS (%)	29.01 ± 8.30	33.53 ± 7.14	0.003
E/LASr/LVEDV ratio (%)	389.44 ± 138.29	278.93 ± 90.17	<0.001
E/e′/LVEDV ratio (%)	17.27 ± 7.07	13.30 ± 4.45	0.001
Left atrioventricular coupling index			
LACI (%)	17.12 ± 6.72	12.28 ± 3.96	<0.001

Values are presented as mean ± standard deviation or number (%). DM, diabetes mellitus; LAVI, left atrial volume index; E, E-wave velocity; e′, e′-wave velocity; LASr, left atrial reservoir function; DWS, diastolic wall strain; LVEDV, left ventricular end-diastolic volume; LACI, left atrioventricular coupling index.

### 3.3 Correlation analysis of ultrasound parameters in DM patients

**Figure 4:**
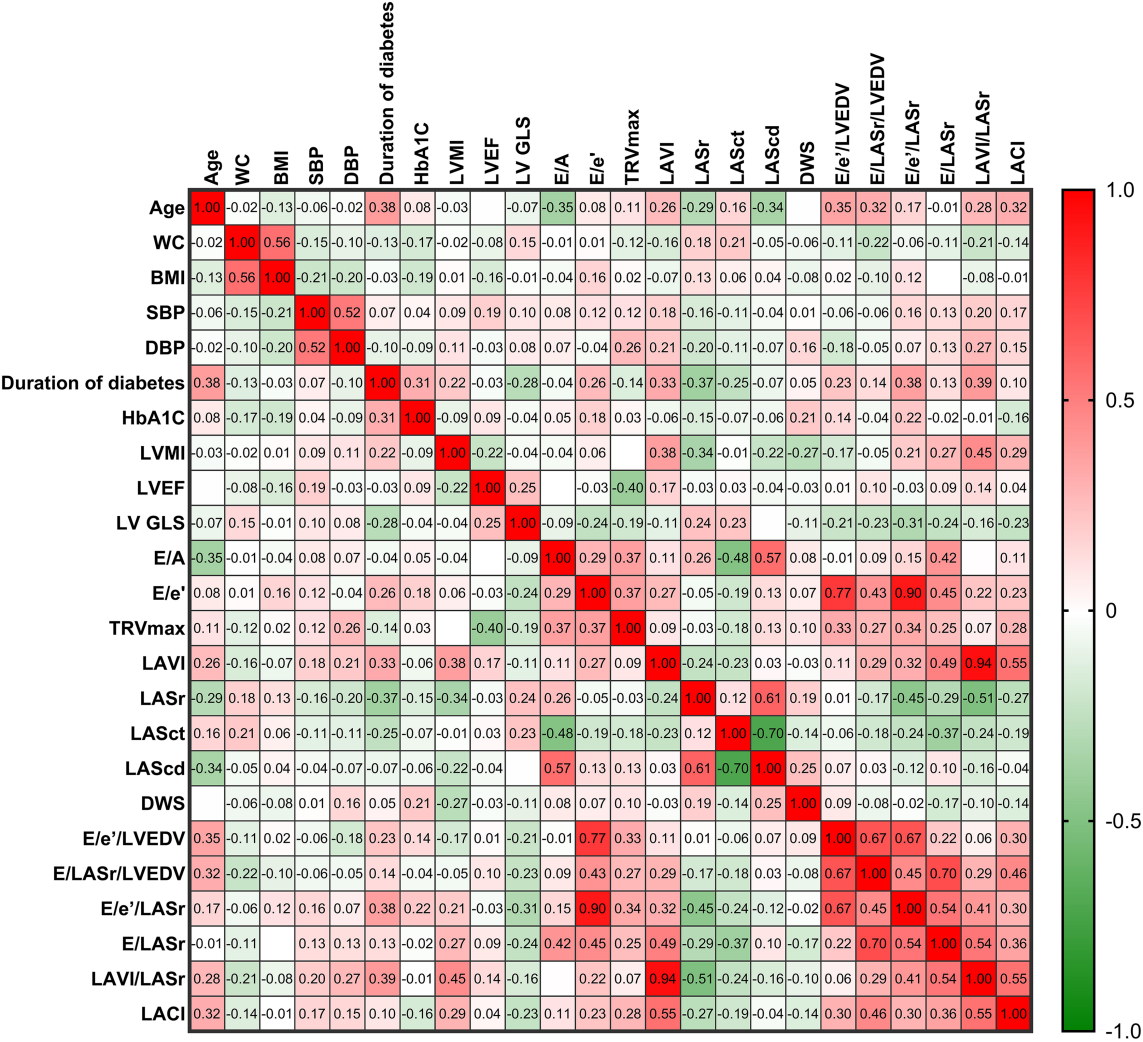
The heatmap illustrates the relationships between LA stiffness indicators, LV stiffness indicators, LACI, and LA and LV parameters in patients with DM. The color shading of each cell corresponds to the magnitude and direction of the correlation coefficient, with red indicating a positive correlation and green indicating a negative correlation. The numerical values within the cells represent the bivariate correlation coefficient. The abbreviations used are the same as those in Tables 1, 2.

The correlations among LA stiffness indicators, LV stiffness indicators, LACI, and LA and LV parameters in patients with DM are illustrated in Figure 4. A positive correlation was observed between E/LASr/LVEDV and E/e′/LASr (*r* = 0.45, *p* = 0.001). The E/LASr showed positive correlations with the E/A ratio (*r* = 0.42, *p* = 0.002), E/e′ ratio (*r* = 0.45, *p* = 0.001), and LV volume index (LAVI) (*r* = 0.49, *p* < 0.001). The LACI was positively correlated with the LAVI (*r* = 0.55, *p* < 0.001). The LACI is also positively correlated with indices of LA and LV stiffness. Additionally, the duration of diabetes mellitus (DM) is positively correlated with both E/e′/LASr and LAVI/LASr. Further details on the specific correlation coefficients can be found in the heatmap (Figure 4).

### 3.4 Logistic regression analysis

**Figure 5:**
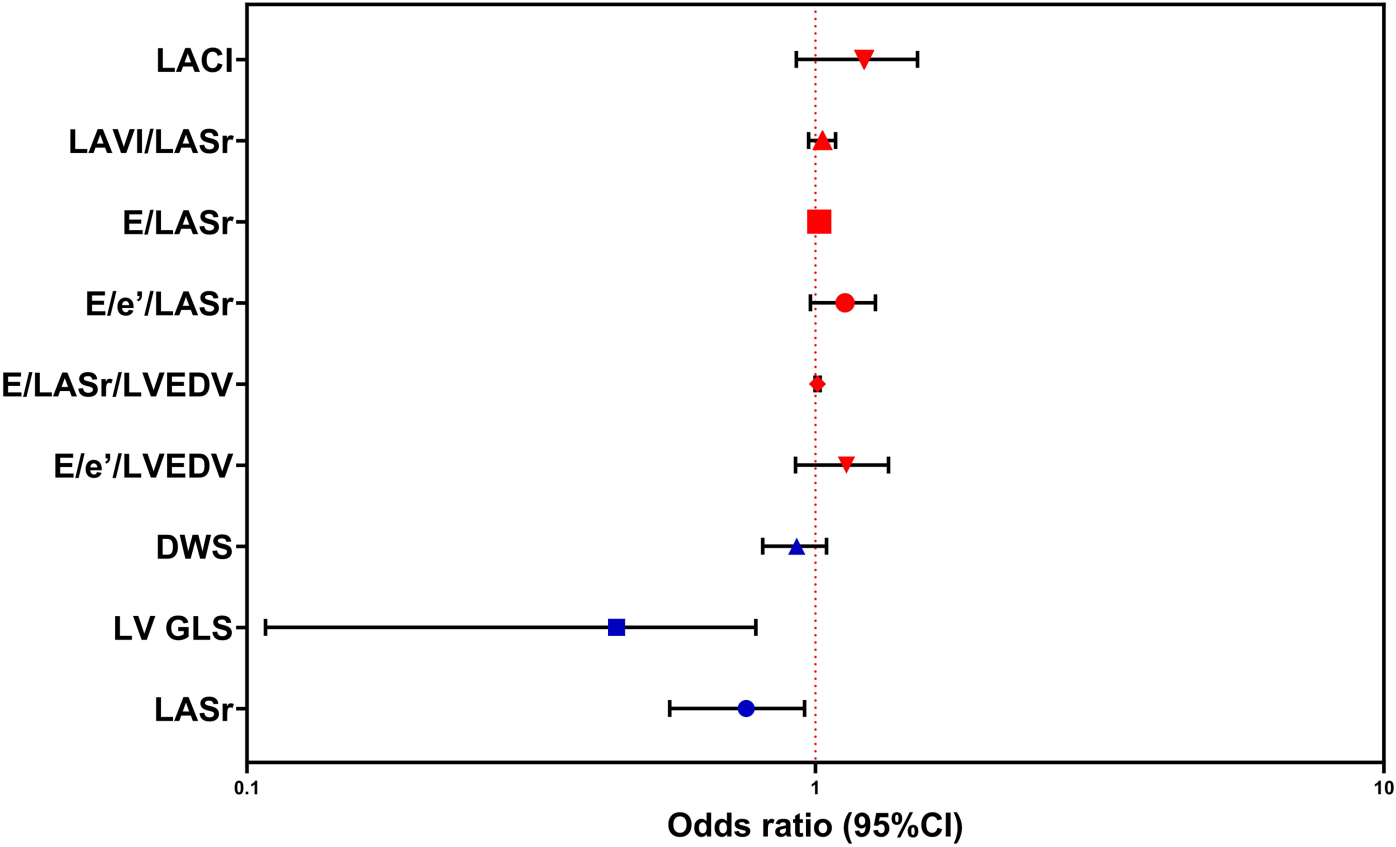
Logistic regression analysis was performed to predict susceptibility to DM, employing LASr, LV GLS, DWS, E/e′/LVEDV, E/LASr/LVEDV, E/e′/LASr, E/LASr, LAVI/LASr, and LACI as covariates. The abbreviations utilized are consistent with those in Tables 1–3.

Table 3 shows the results of logistic regression analyses for LASr, LV GLS, LA stiffness indicators, LV stiffness indicators, and LACI in patients with DM. Decreased levels of LV GLS, adjusted for age, sex, blood pressure, and BMI, have emerged as identified risk factors for DM. Detailed information is clearly depicted in Table 3 and Figure 5.

**Table 3:** Regression analysis for prediction of DM susceptibility.

Variables	Univariable				Multivariable			
	*p*-value	OR	95%CI		*p*-value	OR	95%CI	
LASr	**<0.001**	0.753	0.676	0.838	0.071	0.772	0.582	1.022
LV GLS	**<0.001**	0.433	0.318	0.590	**<0.001**	0.488	0.333	0.714
DWS	**0.001**	1.132	1.050	1.220	0.093	0.928	0.851	1.012
E/e′/LVEDV	**<0.001**	1.009	1.005	1.013	0.433	1.256	0.711	2.221
E/LASr/LVEDV	**<0.001**	1.127	1.069	1.188	0.564	0.992	0.967	1.019
E/e′/LASr	**<0.001**	1.016	1.008	1.024	0.543	0.908	0.664	1.240
E/LASr	**0.010**	1.029	1.007	1.052	0.550	1.014	0.970	1.059
LAVI/LASr	**<0.001**	1.215	1.103	1.339	0.116	0.966	0.925	1.009
LACI	**<0.001**	0.753	0.676	0.838	0.098	1.169	0.971	1.406

Bold values indicate significance *p* ≤ 0.050. OR, odds ratio; CI, confidence interval; DM, diabetes mellitus; LASr, left atrial reservoir function; LV GLS, left ventricular global longitudinal strain; LAVI, left atrial volume index; E, E-wave velocity; e′, e′-wave velocity; LASr, left atrial reservoir function; DWS, diastolic wall strain; LVEDV, left ventricular end-diastolic volume; LACI, left atrioventricular coupling index.

## 4 Discussion

Our study aimed to investigate the impact of DM on LA stiffness, LV stiffness, and LACI using echocardiography. Among the 111 subjects included in the study and divided into DM and control groups, no significant differences were observed in age, sex, BMI, BSA, or WC between the two groups. This selection aimed to minimize the influence of these factors on the assessed cardiac stiffness indices. Furthermore, both the control and DM groups exhibited normal blood pressure ranges, as hypertensive subjects were excluded to eliminate their potential impact on the study (12, 32, 33). Several publications have demonstrated the influence of age and obesity on LA and LV stiffness, supporting our selection criteria (11, 17, 34, 35).

### 4.1 Structural and functional cardiac impairment in individuals with DM

In the comparison of LA parameters using ultrasound, our findings revealed a significant reduction in LA function assessed by STE in the DM group compared to the control group. Similar to our observations, other studies have indicated that DM significantly affects LA function, particularly LA strain (36, 37). Notably, even in patients without abnormal LAVI values, a parameter commonly used in clinical practice (21), changes in LA strain were evident.

Echocardiography of parameters related to the LV indicated a substantial decrease in the LV GLS in DM patients compared to controls. This observation aligns with previous research findings (38, 39). Interestingly, despite no significant difference in LVEF, LV GLS demonstrated alterations in DM patients. This raises questions about whether LV GLS may outperform LVEF in the early detection of LV functional changes in DM patients.

In our study, parameters such as E/e′ and TRVmax, commonly used for assessing LV diastolic function clinically, showed variations between the DM group and the control group. This result is consistent with previous publications (40, 41).

### 4.2 LA stiffness, LV stiffness, and LACI alterations in individuals with DM

Our study indicated that DM patients had greater LA and LV stiffness indicators than did control patients, as clearly illustrated in Table 2 and Figure 3. Various studies have highlighted the impact of DM on cardiac structure and function through multiple mechanisms. These mechanisms include impaired cardiac insulin metabolic signalling, mitochondrial dysfunction, increased oxidative stress, reduced nitric oxide bioavailability, elevated advanced glycation end products, collagen-based cardiomyocyte and extracellular matrix stiffness, impaired mitochondrial and cardiomyocyte calcium handling, inflammation, renin-angiotensin-aldosterone system activation, cardiac autonomic neuropathy, endoplasmic reticulum stress, microvascular dysfunction, and various cardiac metabolic abnormalities. The molecular mechanisms implicated in underlying pathophysiological changes include abnormalities in AMP-activated protein kinase, peroxisome proliferator-activated receptors, O-linked N-acetylglucosamine, protein kinase C, microRNA, and exosome pathways (42).

LA stiffness and LV stiffness have been demonstrated to have prognostic value in predicting cardiac events such as atrial fibrillation and heart failure (9, 43, 44). Our findings suggest that these stiffness indices change in DM patients before alterations in commonly used clinical indices such as the LAVI. This hypothesis implies that LA stiffness and LV stiffness may not only have prognostic value but also serve as early indicators of structural and functional cardiac changes in DM patients.

Despite the independent prognostic value of LA and LV parameters for cardiovascular events, recent research has suggested that incorporating LACI into risk prediction models enhances model discrimination and reclassifies cardiovascular risk. Compared with individual LA parameters, the LACI determines early LA remodelling stages and has greater prognostic value in predicting cardiac events after adjusting for traditional risk factors (17). In our study, the LACI also demonstrated significant alterations in DM patients compared to the control group. Similarly, a recent study in DM and hypertension patients showed an increase in LACI in the DM group (45). The promising prognostic and early detection capabilities of LACI, which can be easily assessed through rapid and straightforward echocardiographic measurements, offer optimistic prospects for widespread clinical application in the future.

### 4.3 Limitations

Despite yielding promising results, our study is not without limitations. First, as a cross-sectional investigation, its focus was on evaluating the impact of DM on LA stiffness, LV stiffness, and the LACI at a single time point. To comprehensively assess the diverse stages of DM, extended follow-up studies are essential. Furthermore, this was a single-center study with a modest sample size exclusively comprising Vietnamese subjects, which limits the generalizability of our findings and may introduce selection bias. To enhance applicability and validity, multicenter studies involving larger cohorts across diverse populations are imperative. Additionally, due to ethical considerations, patients did not cease medication use during sample collection. Moreover, considering the context at our research site, where changes in treatment medications are frequent, we did not exclude the potential influence of antidiabetic medications on cardiac function. The investigation exclusively employs a singular ultrasound machine model and software. Notably, this study did not perform a comparative analysis across different ultrasound machine manufacturers or analysis software for STE. Finally, the observational nature of our data restricts a full elucidation of the mechanisms linking DM with left atrioventricular coupling derangements. While offering valuable insights, causation cannot be inferred. Future research, incorporating experimental or interventional studies, is proposed to unravel these mechanistic associations. Addressing these limitations in subsequent research endeavors will contribute significantly to a nuanced understanding of the intricate relationship between DM and cardiac parameters in the medical field.

## 5 Conclusions

LA stiffness indicators, LV stiffness indicators, and LACI are greater in patients with diabetes mellitus than in healthy individuals. This finding suggests a potential link between diabetes and increased stiffness in both the atrial and ventricular components of the heart.

## Data Availability

The datasets presented in this article are not readily available because the data is unavailable due to privacy and local ethical restrictions. Requests to access the datasets should be directed to Hai Ngoc Nguyen Dang (dangnngochai@dtu.edu.vn or ngochai123dc@gmail.com).
